# Length of stay in a home treatment team

**DOI:** 10.1192/bjo.2021.667

**Published:** 2021-06-18

**Authors:** Mudasir Firdosi, Nicole Gill, Dieneke Hubbeling

**Affiliations:** 1 South West London and St George's Mental Health NHS Trust, St George's, University of London; 2 St George's, University of London; 3 South West London and St George's Mental Health NHS Trust

## Abstract

**Aims:**

The aims were to establish the mean length of stay (LOS) in the Wandsworth home treatment team (HTT), and to identify which variables were associated with LOS. We hypothesised that the variables that are routinely collected via the electronic record system were associated with the LOS.

**Background:**

Psychiatric HTT's have been set up in all NHS trusts in England. These 24-hour community health services exist to assess and manage patients during a crisis, who would otherwise be admitted to an acute psychiatric ward. HTT's also allow inpatients to be discharged sooner, as their treatment can continue in the community. Currently, research into predictors of LOS in HTT's is limited.

Researchers have been exploring whether LOS in psychiatric inpatients can be predicted, but no consistent pattern has emerged. This suggests that LOS is mainly determined by the local service organisation, and the individual circumstances of the patients.

**Method:**

Routinely collected data about all patients under the care of the Wandsworth HTT during the financial year 2018/2019 were used. Only the first admission per individual was considered. Admissions lasting less than 2 days, or more than 42 days were excluded. This is on the basis that those with a very short LOS had not consented to being treated at home, and those with a very long LOS were due to administrative errors. This resulted in a total of 664 admissions being included in the study. The available data for analysis included age, gender, diagnosis, HoNOS cluster, ethnicity, nationality, religion, marital status, referral source, employment status, accommodation status, and accommodation type. The data were analysed in SPSS version 25 using ANOVA, independent samples T-test, and Pearson's correlation.

**Result:**

The mean LOS in the Wandsworth HTT was 14.28 days (standard deviation: 8.57). LOS was positively skewed, with a median LOS of 13 days, but 46.5% of admissions had a LOS longer than this. None of the variables (age, gender, diagnosis, HoNOS cluster, ethnicity, nationality, religion, marital status, referral source, employment status, accommodation status, and accommodation type) had a significant association with LOS, but there was a trend for referral source and accommodation type.

**Conclusion:**

The results from this study suggest that LOS cannot be consistently predicted in the Wandsworth HTT from the routinely collected variables, and that it is the specific circumstances of individual patients that determine their LOS.

There was no external funding for this study.

